# Advanced primary abdominal pregnancy with a live birth: a case report

**DOI:** 10.1515/crpm-2026-0004

**Published:** 2026-08-10

**Authors:** Mourad Elfaham, Ahmed Zeinhom, Mohamed Arafa, Mohamed Essam-Eldin, Ahmed Yaseen, Menatallah Abdelhamid, Nermin Mohamed, Mayar Osama, Romany Alfy, Rahma AbdelHafez, Sara Abdelkader, Mohammed Ghanem, Sally Aboelenin, Mahmoud Amin, Ahmed Abdelkader, Amal Othman, Omnia Seyam, Khaled Eldieb, Eman Rashad, Karam Bayoumy, Ashraf Nabhan

**Affiliations:** Department of Obstetrics and Gynecology, Faculty of Medicine, Ain Shams University, Cairo, Egypt; Department of Obstetrics and Gynecology, Faculty of Medicine, Galala University, Suez, Egypt; Department of Urology, Faculty of Medicine, Ain Shams University, Cairo, Egypt; Department of Anesthesia, Faculty of Medicine, Ain Shams University, Cairo, Egypt; Department of Radiology, Faculty of Medicine, Ain Shams University, Cairo, Egypt; Department of Vascular Surgery, Faculty of Medicine, Ain Shams University, Cairo, Egypt; Department of Pediatrics, Faculty of Medicine, Ain Shams University, Cairo, Egypt; Medical Research Unit, Faculty of Medicine, MTI University, Cairo, Egypt

**Keywords:** advanced abdominal pregnancy, live birth, case report, literature review

## Abstract

**Objectives:**

Advanced primary abdominal pregnancy, an extremely rare condition, poses diagnostic challenges and carries a risk of serious maternal and perinatal complications. In the absence of evidence-based protocols, current management approaches are predominantly guided by anecdotal reports and individual clinical judgment. We report a case of primary advanced abdominal pregnancy in a primigravida.

**Case presentation:**

A 26-year-old asymptomatic woman at 31 + 6/7 weeks’ gestation was referred for routine third-trimester ultrasonography, which revealed a non-gravid uterus, a viable extrauterine fetus, oligohydramnios, and placental invasion of the right pelvic wall. Magnetic resonance imaging confirmed these findings and demonstrated placental compression of the right ureter with moderate hydroureteronephrosis. A multidisciplinary team was assembled. At 32 + 6/7 weeks, cystoscopic-guided ureteric stenting followed by midline laparotomy resulted in the delivery of a live female neonate. Due to extensive placental vascularity and critical attachments, the placenta was left *in situ*, and postoperative methotrexate alternating with folinic acid was administered to promote placental involution. The postoperative course was uneventful. Serial follow-up imaging showed progressive placental regression.

**Conclusions:**

Even when facing a rare, challenging condition, favorable maternal and neonatal outcomes can be achieved through accurate pre-operative diagnosis, early involvement of a multidisciplinary team, thoughtful intra-operative decision-making, and meticulous post-operative care.

## Introduction

### Rarity and definition

Advanced abdominal pregnancy (AAP) is an exceptionally rare obstetric complication, with an estimated incidence of approximately 1 in 10,000 to 1 in 30,000 pregnancies [1], 2]. An “advanced” abdominal pregnancy specifically refers to one that has progressed beyond 20 weeks of gestation. Cases where the pregnancy progresses to a viable live birth are even more rare. Abdominal pregnancy is either primary or secondary. Primary abdominal pregnancies are the less common subtype, as most abdominal pregnancies are secondary, resulting from a tubal abortion or rupture with subsequent re-implantation in the abdominal cavity [2]. Primary abdominal pregnancies are defined as pregnancies attached solely to the peritoneal surface with no involvement of the Fallopian tubes or ovaries and with no uteroperitoneal fistula. Implantation may occur anywhere on the peritoneal surface or abdominal viscera, the most common sites being the uterine pouches (24.3 %), uterine and tubal serosa (23.9 %), or multiple sites (12.8 %) [3], 4].

### Clinical challenges and risks

Advanced primary abdominal pregnancy is a life-threatening condition for both mother and fetus, presenting significant clinical challenges in diagnosis [5] and high risks of serious complications [6], 7]. AAP is difficult to diagnose, with many cases missed on initial ultrasound scans. There is no well-established, standard of care for managing AAP, particularly when diagnosed at a viable gestational age. Management decisions require a highly specialized, multidisciplinary team approach, often in a tertiary care center. The main management challenge is the placenta. Complete removal carries a significant risk of catastrophic hemorrhage, while leaving it *in situ* increases the risk of infection, abscess formation, and secondary hemorrhage, requiring careful case-by-case decisions. The maternal mortality rate is estimated to be approximately 0.5–18 % [8], which is 90 times higher than that of a normal intrauterine pregnancy [2]. Fetal malformations, small for gestational age, and preterm birth contribute to a poor fetal and neonatal prognosis, with fetal and perinatal mortality rates ranging between 40 and 95 % [6], [7], [8].

### Clinical relevance of this case

We report a rare case of advanced primary abdominal pregnancy in a primigravida. Despite the diagnostic challenges and the surgical complexity, a successful management, with favorable maternal and fetal outcomes, was achieved.

### Case presentation

A 26-year-old primigravida at 31 +6/7 weeks of gestation, was diagnosed with an advanced abdominal pregnancy at Ain Shams University Hospital, Egypt. The timeline is detailed in Table 1.

**Table 1:** Patient timeline.

Date	Timeline
November 27, 2024	Admission from the obstetric clinic during routine antenatal visits. Diagnosis of advanced abdominal pregnancy at 31 +6/7 weeks using US and MRI. A multidisciplinary team assembled. The team reviewed the case and planned the operative intervention.
December 3, 2024	In the operative room, the consultant uro-surgeon placed a cystoscopy-guided ureteric stent. Consultant obstetricians delivered a live female newborn (1,450 g) by midline laparotomy. Placenta left *in situ*. The ureteric stent was uneventfully removed immediately after the operation. Patient transferred to intensive care unit for post-operative care, showing stable vital data, adequate urinary output, and clear intraperitoneal drain.
December 4, 2024	Initiation of methotrexate therapy alternating with intramuscular folinic acid over 8 days.
December 5, 2024	Measurement of β-hCG titre was found to be 18,000 mIU/mL.
December 8, 2024	Removal of intraperitoneal drain.
December 9, 2024	Follow-up US revealed a right adnexal pelviabdominal heterogeneous placenta with signs of placental invasion, no fluid collections, and improvement of previously noted hydronephrosis. Discharge from the intensive care unit (ICU).
December 16, 2024	Measurement of β-hCG titer showed reduction to 3,400 mIU/mL. Follow-up US showed decreased placental size.
December 18, 2024	Discharge from hospital.
January 10, 2025	Follow-up US showed decreased placental vascularity.
January 17, 2025	Readmission to receive second course of methotrexate/folinic acid. Onset of low grade fever for 2 days after the initiation of methotrexate regimen.
January 22, 2025	Follow-up contrast MRI of the abdomen and pelvis.
February 1, 2025	Completion of methotrexate and folinic acid therapy Discharge of patient.
February 25, 2025	Follow-up ultrasound with color mapping showed normal pattern of placental tissue measuring 13 × 14 cm, with encysted adhesions, and no visualized vasculature.
April 7, 2025	Follow-up ultrasound revealed normal pattern of placental tissue along with encysted turbid collection 11 × 8 cm.
April 8, 2025	Follow up laboratory workup showed normal blood picture and kidney function tests.
May 27, 2025	Follow-up ultrasound showed normal pattern of placental tissue, measuring 13 × 8.7 cm, closely related to the right internal iliac vessel.

Earlier in her antenatal care, she had an ultrasound suggesting a diagnosis of placenta previa. She had no significant past medical history. On November 27, 2024, she was referred from the antenatal clinic for a routine follow up ultrasound.

**Figure 1:**
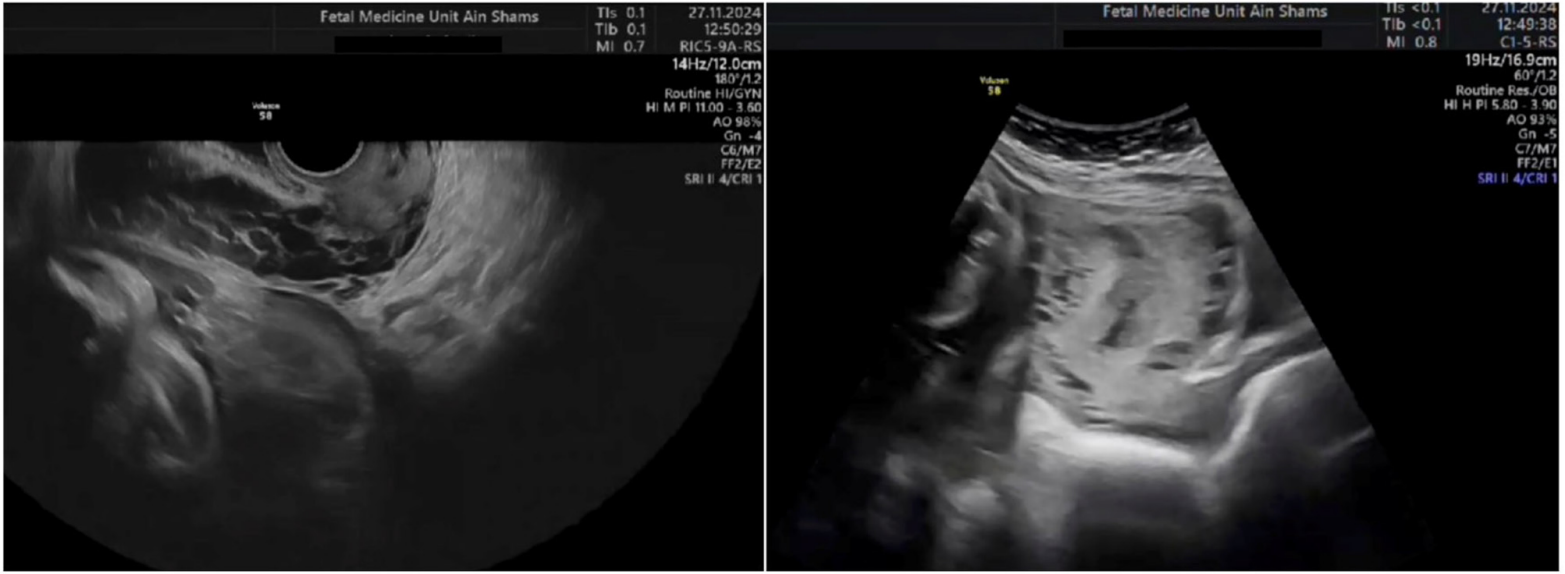
An ultrasound showing a non-gravid uterus, an extrauterine viable fetus.

The ultrasound showed a non-gravid uterus, an extrauterine viable fetus corresponding to 28 +6/7 weeks’ gestation with an estimated fetal weight (EFW) of 1,222 g, oligohydramnios, and signs of placental invasion to adjacent structures on the right pelvi-abdominal wall (Figure 1).

**Figure 2:**
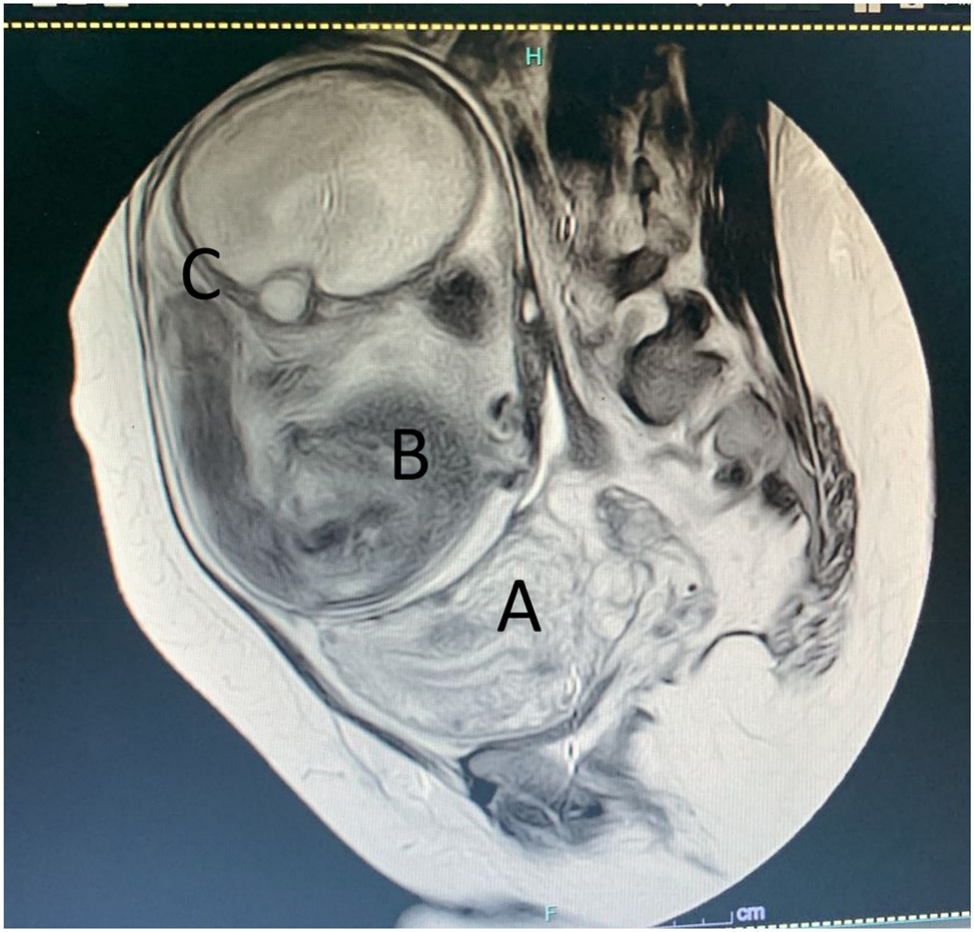
Magnetic resonance imaging showing (A) intact uterus, (B) placenta, (C) extrauterine fetal sac with viable fetus.

An urgent magnetic resonance imaging (MRI) confirmed an empty uterus and a large fetal sac lying superior to the urinary bladder with no direct attachment to and not covered by myometrium. The right uterine artery was seen in continuity with the feeding vessels of placenta. A remarkable compression of the right ureter by the posterolateral aspect of the placenta at the level of the fourth lumbar vertebra resulted in moderate hydroureteronephrosis. The distal segment of the right ureter was poorly visualized. A localized septated collection was noticed lying posterior to the placenta on its right side measuring about 5.8 × 4.8 × 5.6 cm, suggesting a subacute hemorrhage. The uterine wall appeared displacing the left lateral aspect of the urinary bladder downwards and to the right (Figure 2).

In a multidisciplinary approach, senior consultants from urology, general surgery, and vascular surgery were notified and were involved in planning and executing the management.

Following full pre-operative workup, an exploratory lapartomy was performed on December 3, 2024.

**Figure 3:**
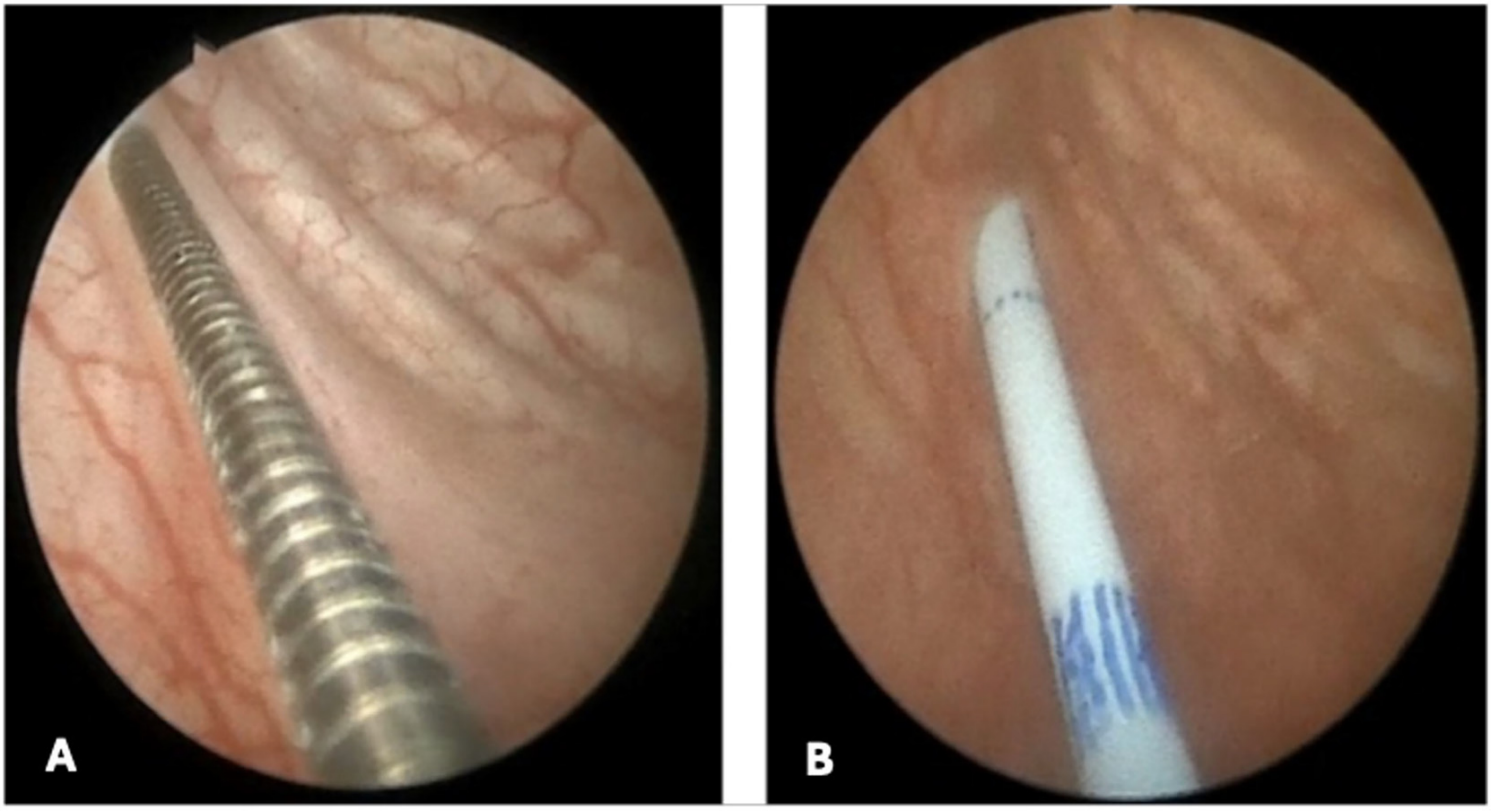
Pre-operative cystoscopic-guided ureteric stent. (A) Guide wire inserted. (B) Ureteric stent inserted.

Pre-operatively, the urosurgeon placed a cystoscopic-guided ureteric stent which was uneventfully removed immediately at the end of laparotomy (Figure 3).

**Figure 4:**
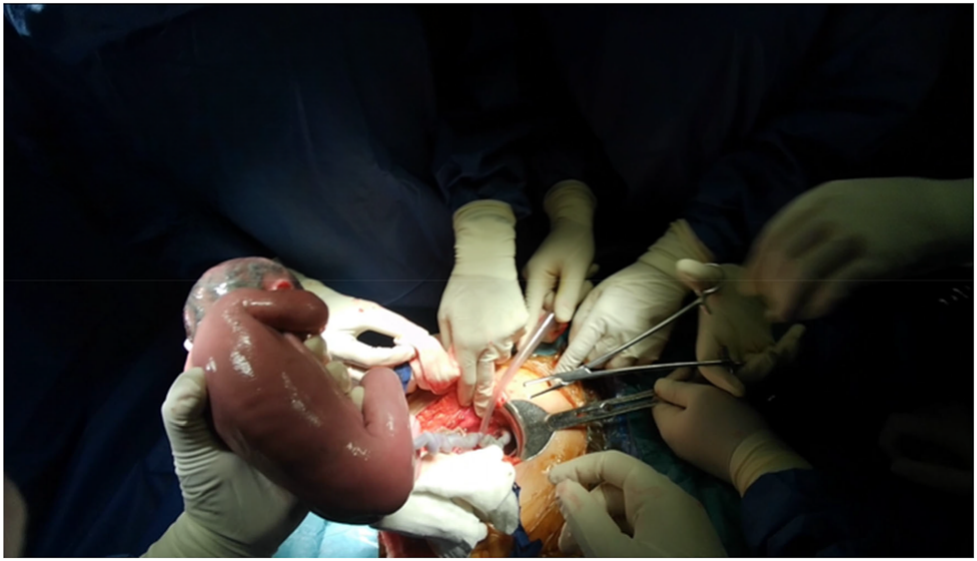
Midline laparotomy and extraction of a single live female, weighing 1,450 g from the abdominal cavity.

A midline laparotomy revealed a gestational sac in the abdominal cavity. A single live female newborn, weighing 1,450 g, was extracted, Figure 4. The newborn had an APGAR score of five at 1 min and eight at 5 min, and had no apparent malformation or deformities.

**Figure 5:**
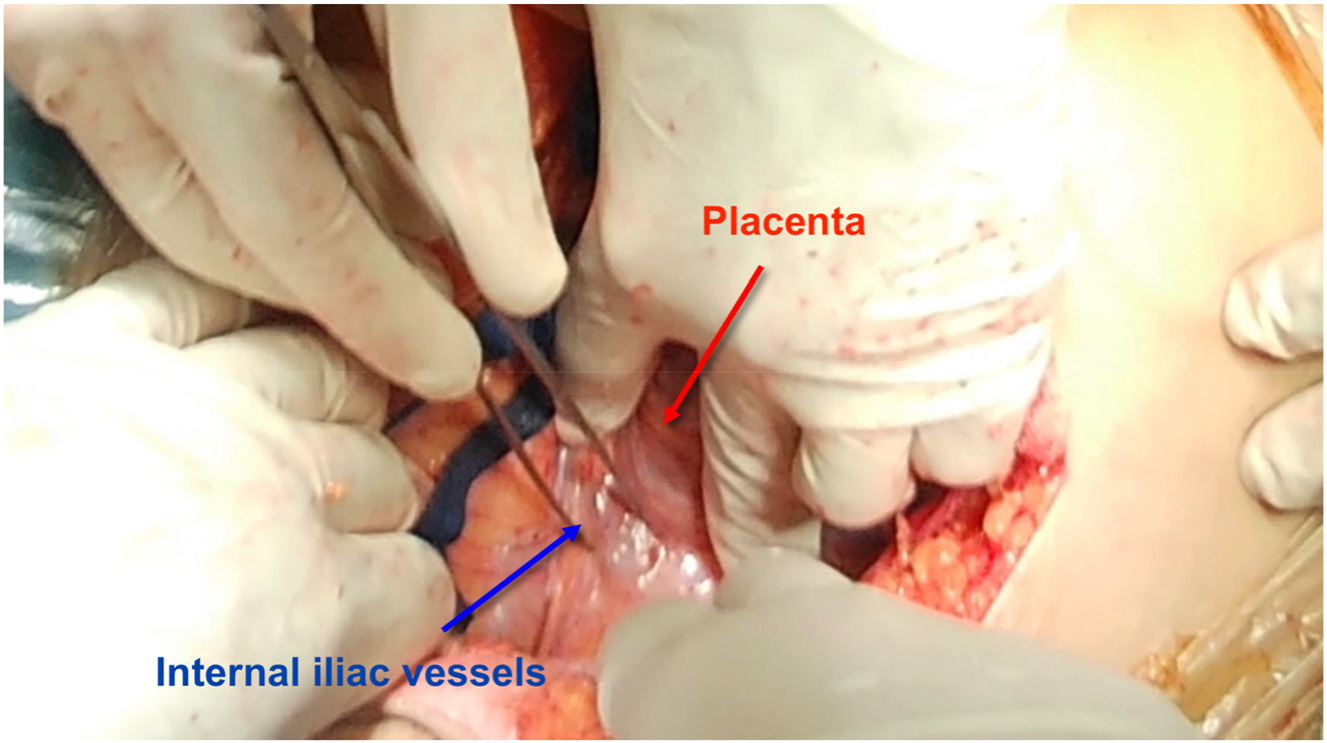
Exploring site of placental implantation. Placenta (red arrow) and internal iliac artery (blue arrow).

The placenta was left *in situ* following a second opinion from the head of the unit and the vascular surgeon, Figure 5. Hemostasis was ensured, and an intra-peritoneal drain was inserted. An unedited video of the full operative management is available in the Supplementary File.

The patient was transferred to the intensive care unit (ICU) for postoperative care, and, upon admission, the patient’s vitals were monitored and found to be stable: blood pressure was 120/80 mmHg, pulse was 90 bpm, and temperature was 37.2 °C. The intraperitoneal drains showed no output. Urine output was adequate (100 mL/h).

On the first postoperative day, December 4, 2024, the patient started a methotrexate regimen of 1 mg/kg per dose on days 1, 3, 5, and 7 alternating with intramuscular folinic acid on days 2, 4, 6, and 8. On measuring her β-hCG titre on December 5, it was found to be 18,000 mIU/mL. On December 8, the intraperitoneal drain was removed. A follow-up ultrasound on December 9 revealed a right adnexal pelviabdominal heterogeneous placenta measuring approximately 14 × 9 cm, with signs of placental invasion. No fluid collections were detected in the hepatorenal angle or Douglas pouch, and the previously noted hydronephrosis had significantly improved. The patient was discharged from the intensive care unit (ICU) on December 9.

A follow up β-hCG titre was performed on December 16 and showed a marked reduction to 3,400 mIU/mL, along with a decrease in placental size to 13 × 9 cm and improvement in the previously noted hypervascularity. The patient was discharged from the hospital on December 18, 2024.

On January 10, 2025, a follow up ultrasound was performed, revealing a normal pattern of placental tissue measuring 13 × 11 cm and significant decrease in vascularity. There were also noticeable encysted adhesions measuring 10 × 8 cm.

The patient was readmitted to the hospital on January 17, 2025, to receive a second course of intramuscular methotrexate alternating with intramuscular folinic acid over 8 days. She experienced a low -grade fever after starting the methotrexate regimen, which lasted for 2 days.

On January 22, 2025, MRI of the abdomen and pelvis with contrast was conducted, in which the remnant sac was seen in the lower abdomen and pelvic region, collectively measuring 10.7 × 17.2 × 16.3 cm. This sac contained a large heterogeneous placenta with a stationary course compared to the previous study. It demonstrated patches of high T1 signal denoting thrombosis/hemorrhage and areas of low T2 signal suggesting involuted tissue. The rest of the remnant sac contained high T1 fluid, likely representing hemorrhagic content.

The placental tissue exhibited peripheral tortuous feeding vessels, which originated from the right internal iliac artery. Additional vascular supply was noted from the uterine artery and cervix on the right side, as well as from the right anterolateral abdominal wall. These findings indicated a persistently vascularized remnant.

The placenta abutted the superior aspect of the right ovary and compressed the right ureter, mostly at its middle segment, at the level of the L4 vertebra. This resulted in moderate right-sided hydroureteronephrosis, and the distal part of the right ureter was barely visualized. The placenta laid adjacent to the urinary bladder, without displacing bowel loops or showing signs of infiltration.

The uterus appeared normal in position and size, with regular endometrial thickness and no myometrial focal lesions. Both adnexa were unremarkable with no focal masses. The urinary bladder was mildly displaced downward and to the right, but its wall thickness remained normal, and there were no stones or masses. The left internal iliac vessels demonstrated normal appearance.

The abdominal organs were also evaluated. The liver showed homogenous parenchymal signals with no focal lesions and no biliary ductal dilation; the portal vein was patent. The gallbladder showed no wall abnormalities or stones. The spleen and pancreas appeared normal without focal lesions. Both kidneys had normal size and shape, with no stones or hydronephrosis; however, simple renal cysts were noted bilaterally, without causing left-sided back pressure. No ascites or enlarged abdominal or pelvic lymph nodes were identified.

The patient completed the course of methotrexate and was discharged on February 1, 2025. Then, a follow up ultrasound was performed on February 25, showing a normal pattern of placental tissue with encysted adhesions measuring 13 × 14 cm with color mapping and no visualized vasculature. Another follow up ultrasound with color mapping was performed on April 7 and revealed a normal pattern of placental tissue measuring 12 × 12 cm and minimal vasculature, along with encysted turbid collection measuring 11 × 8 cm. On April 8, work up labs were ordered revealing normal blood picture and kidney function tests. On May 27, 2025, a follow up ultrasound with color mapping was performed and showed a normal pattern of placental tissue measuring 13 × 8.7 cm and minimal vasculature. The placental tissue was noted to be seen closely related to the right internal iliac vessel. The patient recovered favorably without sequelae and returned to her normal daily activities. A timeline summary covering the pre-operative, intra-operative, and post-operative follow-up periods is provided in Table 1.

## Literature review

A systematic search of MEDLINE was performed in November 2025 using the terms “Abdominal pregnancy” AND “live birth” with no language restrictions. Reference lists of eligible reports were also screened. The search yielded only four cases of advanced primary abdominal pregnancy in primigravidas (Table 2) [9], [10], [11], [12].

**Table 2:** Characteristics of four cases of primary advanced abdominal pregnancy reported with favorable maternal and fetal outcomes.

Author year	Country	Pre-operative diagnosis	Operative findings	Gestational age at delivery, weeks	Birth weight, g	Neonatal outcome	Maternal outcome
Than 2023 [9]	Malaysia	None Admitted for elective cesarean section due to transverse lie.	A live male, delivered via a right paramedian incision. Uterus and adnexa were normal. The placenta was implanted on the greater omentum and required omentectomy for complete removal.	38	3,062	Uneventful with no congenital anomalies	Uneventful recovery, discharged on day 7.
Nassali 2016 [10]	Botswana	None Admitted at 41 weeks; failed labor with non-reassuring fetal heart rate necessitated a cesarean section.	Live male delivered. Intact sac with grade 3 meconium. Uterus and adnexa normal. Placenta attached to omentum, ileal mesentery, and Douglas pouch. Placenta delivered following partial detachment that caused bleeding and required blood transfusion.	41	3,108	No congenital anomalies; neonatal death on day 2 from respiratory distress/meconium aspiration.	Uneventful recovery. Ambulant by day 2. Transient ileus resolved. Discharged on day 7.
Paluku 2020 [11]	Democratic Republic of Congo	None At 33 weeks, presented with severe abdominal pain; low-lying placenta and oligohydramnios. Emergency laparotomy was done for suspected peritonitis.	A live female delivered via midline laparotomy. Uterus and adnexa normal. Placenta, attached to omentum and small-bowel mesentery, was left *in situ*.	33	2,000	Uneventful with no congenital anomalies	Uneventful recovery, discharged without complications.
Ramachandran 2004 [12]	United Kingdom	None Admitted at 38 weeks for cesarean due to persistent oblique lie.	Live male delivered via Pfannenstiel. Placenta partly removed from bowel/mesentery. Ongoing instability required a second laparotomy and ICU care.	38	3,130	Uneventful with no congenital anomalies	Wound infection and hematoma required re-operation and a 3-week stay. MRI showed residual placenta, recovered well.

Maternal age ranged from 24 to 32 years. The gestational age at delivery ranged from 33 to 41 weeks. None of the previously reported cases were successfully diagnosed preoperatively, highlighting the diagnostic challenges of primary abdominal pregnancy. The most common presentation was persistent abdominal pain. One case was asymptomatic [10].

Reported maternal complications included transit paralytic ileus [10], severe hemorrhage up to hemorrhagic shock, wound infection, and hematoma [12]. One case reported fetal respiratory distress due to meconium aspiration, resulting in neonatal death [10]. All other neonates were born healthy with no complications.

Management of the placenta varied among cases. In two cases, the placenta was completely removed [9], 10]. In one of these two cases, partial omentectomy was required to remove an adherent placenta [9]. In the third case, the placenta was left *in situ* to mitigate potential intra-operative bleeding [11]. In the fourth case, partial removal of placenta was performed [12].

## Discussion

We present a meticulous review of cases, specifically of primary advanced abdominal pregnancy. We also present a primary advanced abdominal pregnancy diagnosed preoperatively in an asymptomatic primigravida, and managed by a multidisciplinary team achieving a favorable maternal and perinatal outcome.

Abdominal pregnancy is an extremely rare and life-threatening condition. Being primary is even more rare. The vast majority of abdominal pregnancy are secondary to a ruptured rudimentary uterine horn [13], 14], ruptured tubal pregnancy, ruptured cornual/interstitial pregnancy, or heterotopic pregnancies [15]. Almost all of the reported risk factors for abdominal pregnancy are associated with the secondary subtype. Risk factors include prior ruptured ectopic [16], polycystic ovaries syndrome [17], history of pelvic inflammatory disease, Intrauterine device, and assisted reproductive technology such as *in vitro* fertilization (IVF) [18], [19], [20]. A history of tubal surgery, mainly bilateral salpingectomy, was found in 50 % of abdominal pregnancy following IVF [18], 19].

Women with abdominal pregnancies commonly present with nonspecific symptoms, including persistent abdominal pain, vaginal bleeding, painful fetal movements, malaise, nausea, and vomiting [16], 21]. Physical findings include abdominal tenderness, easily palpable fetal parts, malpresentation [9], and displaced uterine cervix [16], 21], 22]. Some patients may also present with severe conditions, such as hemodynamic compromise, uterine prolapse, or preeclampsia [17], [23], [24], [25]. Our presented case remained asymptomatic throughout her pregnancy. A previous case report of an undiagnosed advanced abdominal pregnancy continued beyond 41 weeks of gestation despite routine antenatal care and serial ultrasound reports [10].

The most notable maternal complication is obstetric hemorrhage, occurring in more than 50 % of patients, which can lead to hysterectomy [6], 26]. Other reported complications include hemoperitoneum [17], 27], postoperative ascites, and intestinal obstruction [28]. Our presented case experienced hydroureteronephrosis, localized septate collection, and hypervascularity.

The fetal and perinatal mortality are high [6], [7], [8]. Even among survivors, major morbidity occurs with malformations and deformations in up to 21.4 % of newborns [29]. The most commonly observed defects include facial and/or cranial asymmetry, joint abnormalities, limb deficiencies, and central nervous system malformations [23], 29]. We did not find any evidence of deformity or malformation after meticulous assessment by the neonatology team.

While our presented case was diagnosed antenatally, abdominal pregnancies are often discovered intra-operatively [6], highlighting the need for a high index of clinical suspicion and the appropriate use of necessary imaging. Ultrasound has long been the first line imaging modality. The most consistent ultrasound finding among AAP patients was uterine separation from the fetus, followed by extrauterine placenta and oligohydramnios, all of which were present in our case. Other possible ultrasound features include abnormal fetal lie, poor visualization of the placenta, and impaired fetal visualization by maternal bowel gases [30]. Magnetic resonance imaging has emerged as a potential alternative, due to its increased detail and soft tissue contrast. Besides confirming the diagnosis, MRI showed the exact materno-fetal anatomical relationships, and reveal vascular and placental organ invasion, making them ideal for preoperative planning [31], 32]. Among the MRI findings in our case was the compression of the right ureter by the placenta, which explains the hydroureteronephrosis suffered by our patient. Post-operative MRI may also be used in cases like ours where the placenta had been left in place, in order to monitor response to therapy and assess the condition of the surrounding abdominal organs.

Surgical management was the consistent approach used among advanced abdominal pregnancy, including our presented case, which was delivered by laparotomy at 32 +6/7 weeks of gestation. Whether or not to remove the placenta remains an intra-operative challenge. The majority of authors suggest a complete removal if blood supply can be identified and safely ligated [6], 33].

However, in cases such as the present one, where the placenta extensively involves adjacent structures and placental removal is associated with a substantial risk of catastrophic hemorrhage, leaving the placenta *in situ* with subsequent medical management may represent the safest approach. The use of methotrexate in this setting remains controversial, as some authors have cautioned against its administration because of the potential for rapid placental necrosis, secondary infection, and related complications [33].

All available management options, including interventional radiology and methotrexate therapy, were carefully evaluated by the multidisciplinary team. Following radiological assessment, the consultant radiologist advised against vascular intervention because the placental tissue was supplied by multiple tortuous peripheral feeding vessels arising from the right internal iliac artery, the uterine artery, and the right anterolateral abdominal wall, making embolization technically challenging and potentially ineffective. Consequently, methotrexate was selected as the preferred management strategy because it was readily available, minimally invasive, and had demonstrated favorable outcomes in the limited number of reported cases. Therefore, methotrexate therapy was initiated with close clinical and radiological surveillance.

We used sequential administration of methotrexate and intramuscular folinic acid over 8 days. Monthly follow-up ultrasounds showed progressive reduction in placental size and vascularity, along with a serial decrease in β-hCG.

This case report represents one of the few reported cases of primary advanced abdominal pregnancy diagnosed antenatally in a primigravida with favorable surgical, maternal, and neonatal outcomes. The cornerstones of management include reaching a correct preoperative diagnosis, engaging in early multidisciplinary planning, and exercising meticulous intra-operative judgment to ensure optimal maternal and neonatal outcomes despite the complexity of the surgical procedure and the challenging post-operative management.

## Supplementary Material

Supplementary Material
