# Supplementary material for: Advanced primary abdominal pregnancy with a live birth: a case report
Source: Case Rep Perinat Med. 2026 Aug 10;15(1):20260004. doi: 10.1515/crpm-2026-0004 (PMC13454813; doi:10.1515/crpm-2026-0004)
Supplement: Supplementary file 1 [file j_crpm-2026-0004_suppl_001.pdf]

Supplementary Material

Operative laparotomy video.

The supplementary video can be accessed via the link below:

https://drive.google.com/file/d/1glCBxPwLHzxzjdB-W--_qadNC0dKAtcY/view?usp=sharing
